# Supplementary material for: A control engineering perspective on the advantages of efference copies
Source: Biol Cybern. 2025 Dec 11;120(1):1. doi: 10.1007/s00422-025-01027-z (PMC12698751; doi:10.1007/s00422-025-01027-z)
Supplement: Supplementary file 1 — (pdf 656 KB) [file 422_2025_1027_MOESM1_ESM.pdf]

Supplementary information: A control engineering perspective on the advantages of efference copies

Benjamin P Campbell, Huai-Ti Lin, Holger G Krapp

September 29, 2025

Imperial College London, Exhibition Rd, South Kensington, London, SW7 2BX
E-Mail: b.campbell21@imperial.ac.uk

1 Disturbance rejection

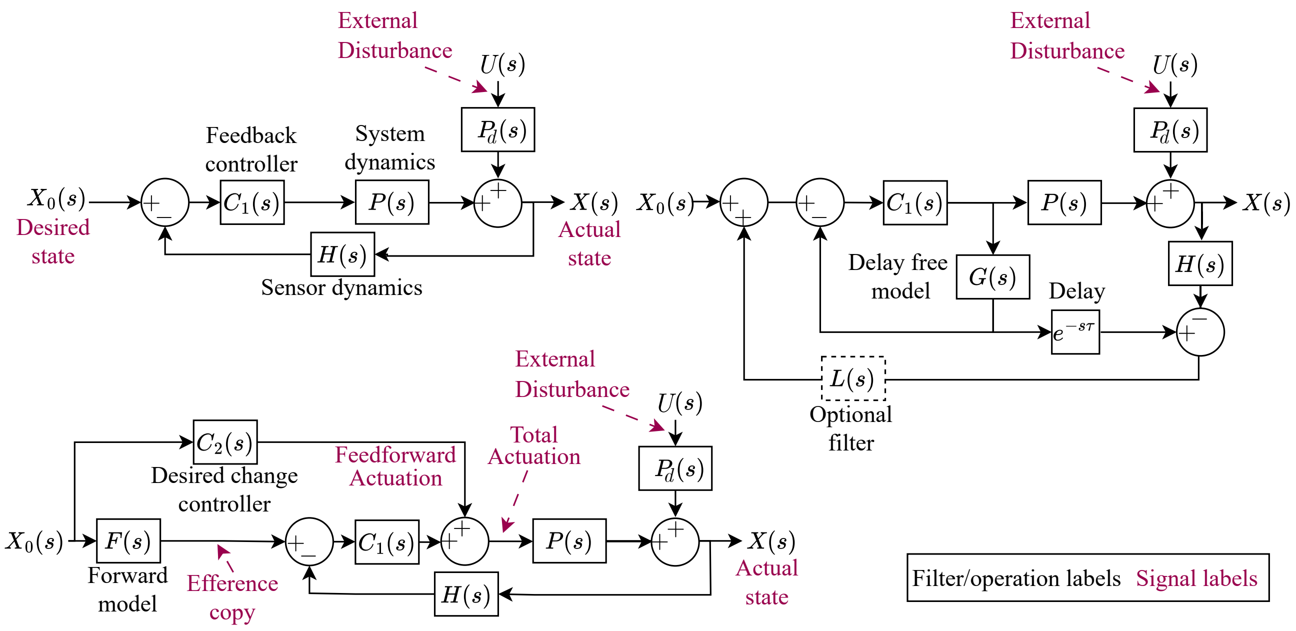

Figure 1: Same as Figure 1 in the manuscript, but the disturbance model is included.

Figure 1 shows how the disturbances were modelled to enter the system in the three architectures compared. The derivations for how the external disturbance affects the state are given below.

FSDoF

$$X(s)\big(1 + HC_1P\big) = X_0(s)\big(C_2P + FC_1P\big) + P_d(s)U(s) \quad (1)$$

$$\text{Set: } X_0(s) = 0 \quad (2)$$

$$X(s)\big(1 + HC_1P\big) = P_d(s)U(s) \quad (3)$$

$$\frac{X}{U}(s) = \frac{P_d}{1 + HC_1P}(s) \quad (4)$$

SP

$$X(s) = P(s)C_1(s)\frac{X_0(s) - H(s)X(s)}{1 + G(s)C_1(s) - e^{-s\tau}G(s)C_1(s)} + P_d(s)U(s) \tag{5}$$

$$\text{Set: } X_0(s) = 0 \tag{6}$$

$$X(s) = \frac{PC_1HX}{1 + GC_1 - e^{-s\tau}GC_1}(s) + P_d(s)U(s) \tag{7}$$

$$\frac{X}{U}(s) = \frac{P_d}{1 + \frac{HCP}{1+GC_1-e^{-s\tau}GC_1}}(s) \tag{8}$$

$$\text{Assume: } GC_1 - e^{-s\tau}GC_1 \approx 0 \tag{9}$$

$$\frac{X}{U}(s) = \frac{P_d}{1 + HCP}(s) \tag{10}$$

PFB

$$X(s)\big(1 + HC_1P\big) = X_0(s)\big(C_1P\big) + P_d(s)U(s) \tag{11}$$

$$\text{Set: } X_0(s) = 0 \tag{12}$$

$$X(s)\big(1 + HC_1P\big) = P_d(s)U(s) \tag{13}$$

$$\tag{14}$$

2 Controller structure and tuning

2.1 FSDoF tuning

To allow for a fair comparison it was important to optimise $F(s)$ and $C_2(s)$ with a single degree of freedom, i.e. one value which we call q. Two things should be satisfied for a legitimate FSDoF controller in the context of these simulations:

$$F(s) = C_2PH(s) \tag{15}$$

$$\lim_{s\to 0} F(s) = 1 \tag{16}$$

$H(s) = 1$ in all the simulations, and assuming the plant dynamics were of the form:

$$P(s) = \frac{P_n(s)}{P_d(s)}e^{-\tau s} \tag{17}$$

We then take the roots of the numerator polynomial such that:

$$P_n(s) = P_n^s(s)P_n^u(s) \tag{18}$$

Where $P_n^s(s)$ and $P_n^u(s)$ are the polynomials of all the negative zeros and positive zeros of the numerator respectively. Substituting this provides:

$$F(s) = C_2\frac{P_n^sP_n^u}{P_d}e^{-\tau s} \tag{19}$$

By letting:

$$C_2(s) = \frac{P_d}{\left(\frac{s}{q} - 1\right)^2 P_n^s} \tag{20}$$

$$F(s) = \frac{P_n^u}{a\left(\frac{s}{q} - 1\right)^2}e^{-\tau s} \tag{21}$$

Where:

$$a = \lim_{s\to 0} P_n^u(s) \tag{22}$$

We can meet the two requirements in Equation 15 and 16, ensure $F(s)$ and $C_2(s)$ are internally stable, and most importantly the FSDoFs reference tracking performance is controlled by a single variable q.

2.2 Controller values

Table 1: Table of controller parameterisations. The Smith predictor had the same gains as the PFB when $\tau = 0$ for all values of τ.

Plant structure	ω (rad/s)	τ (s)	PFB: K_p	PFB: K_i	PFB: K_d	FSDoF: q
1	10	0	1.7212	19.5871	0	37.6369
1	10	0.1	0.86007	5.6566	0	28.9758
1	10	0.05	1.2761	11.2087	0	40.9076
1	50	0	1.7212	97.9357	0	187.825
1	50	0.1	0.40866	7.3928	0	100.4709
1	50	0.05	0.42975	13.1689	0	100.5642
2	10	0	21.2534	6.4618	1.6781	43.0316
2	10	0.1	5.0023	0.42403	0.45127	22.1563
2	10	0.05	9.2896	1.5187	0.99159	32.8344
2	50	0	106.2671	161.5462	1.6781	126.7761
2	50	0.1	7.3683	0.97547	0.3081	44.9396
2	50	0.05	12.8405	2.9942	0.41317	54.7867
3	10	0	1.9691	10.5361	0.014843	18.6125
3	10	0.1	1.4511	6.1648	0.085395	31.5524
3	10	0.05	2.1159	9.1138	0.10817	35.8477
3	50	0	1.9691	52.6805	0.0029686	75.6006
3	50	0.1	0.43646	6.7586	0.0051959	52.8529
3	50	0.05	0.51857	11.5895	0.0051033	53.141
4	10	0	0.16939	56.601	0	63.7718
4	10	0.1	0.8655	8.7584	0	76.3081
4	10	0.05	1.2446	22.0426	0	263.9988
4	50	0	0	0.99964	0	20
4	50	0.1	0	0.99964	0	20
4	50	0.05	0	0.99964	0	20
5	10	0	-7.1377	-40.482	0	206.5766
5	10	0.05	-1.5703	-2.2557	-0.072806	337.1645
5	50	0	-7.1377	-202.4101	0	1031.6281
6	10	0	0	1.0905	0	9
6	10	0.1	0	1.0005	0	9
6	10	0.05	0	0.99738	0	9
6	50	0	0	1.3688	0	49
6	50	0.1	0	1.0093	0	49
6	50	0.05	0	1.3153	0	49

3 Plant structure 6 and non-minimum phase systems

For the non-minimum phase system in plant 6, P_n^u is a single-order polynomial. This means there is an initial undershoot of 0 in the state before settling at the desired state. Figure 2 demonstrates that adjusting q allows you to balance undershoot and settle time.

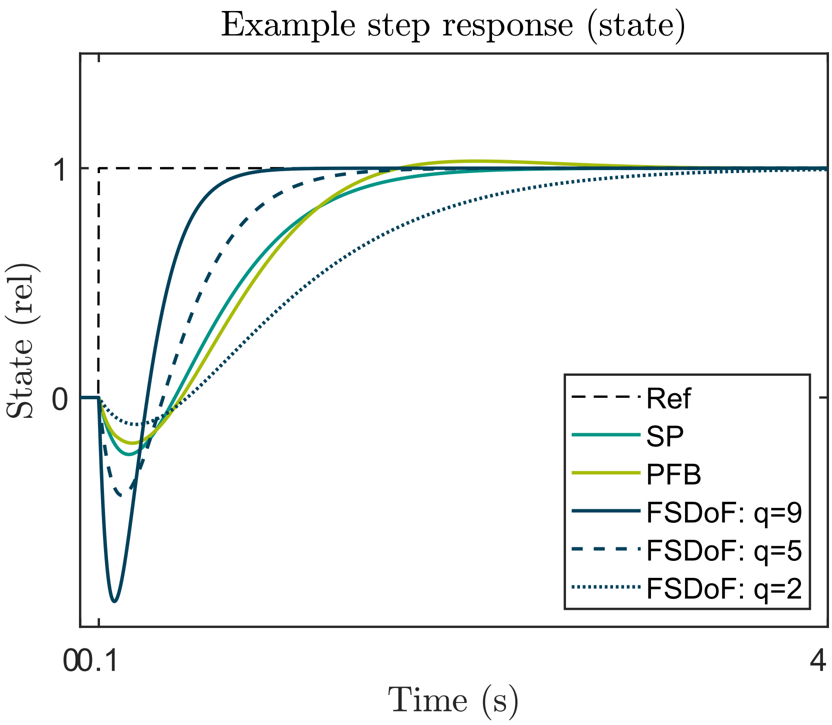

Figure 2: Step response of the SP, PFB, and three FSDoF controllers with different values of q. As q is increased the undershoot increases, but the settle time reduces. This was plant structure 6 with $\omega = 10$ rad/s and $\tau = 100$ ms.

4 Arbitrarily fast settle time of the FSDoF

As explained in the manuscript, unlike the PFB and SP, the FSDoF can settle arbitrarily quickly by increasing q when there is no limit on the maximum actuation available. Examples of this are shown in Figure 3, where the initial value of q is shown, and a simulation with q increased by a factor of 5 is plotted. The only time there is a significant trade-off, not counting increased actuation, is for plant number 6 as shown above where the undershoot dramatically increases.

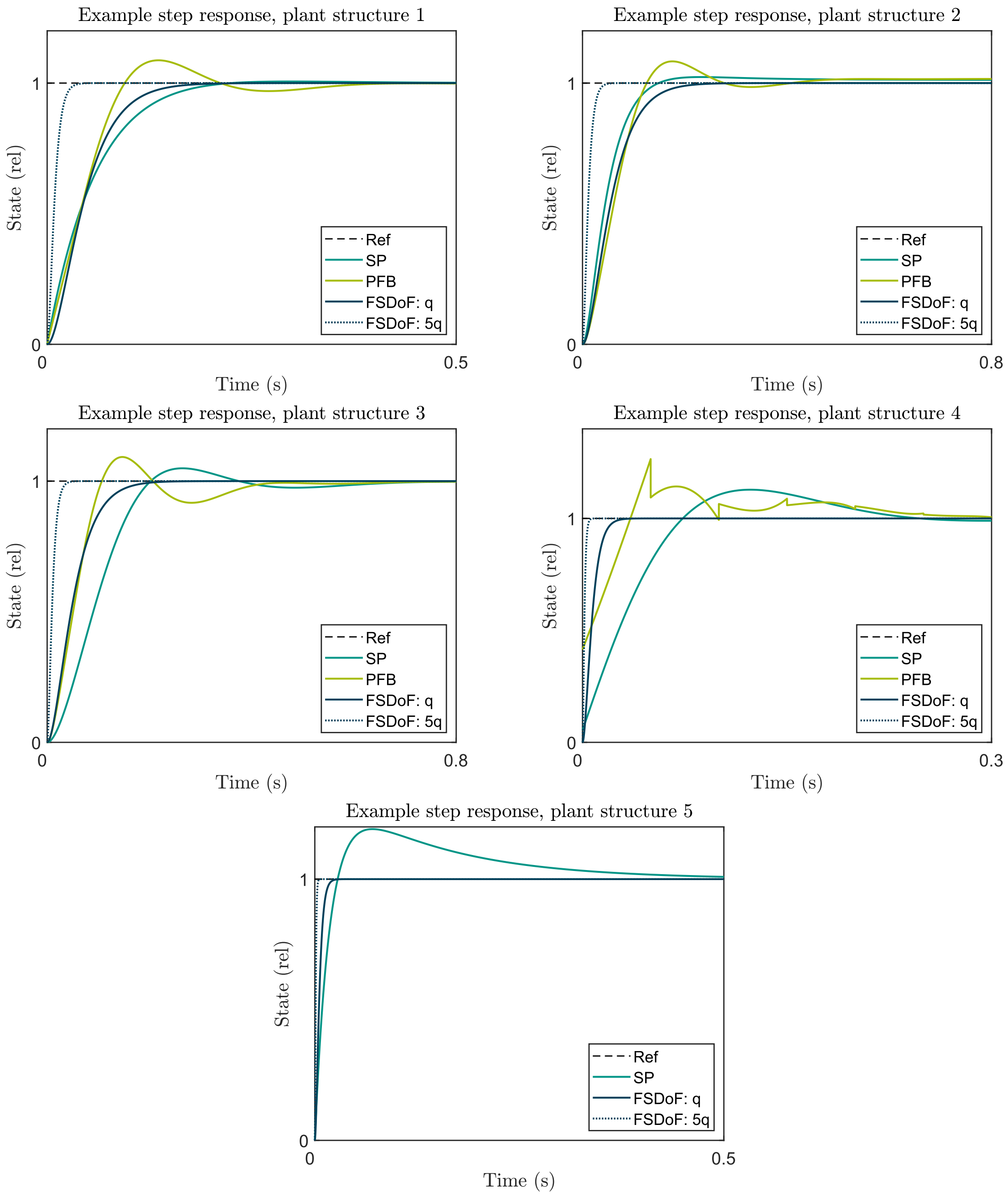

Figure 3: Demonstrating how q can be arbitrarily increased for a faster settle time. Each plant structure is parameterised by $\tau = 50$ ms and $\omega = 10$ rad/s. The example for plant 5 does not include the PFB because it was significantly worse and made the FSDoF results difficult to see when scaled.

5 Results tables

Table 2: Pure feedback controller (PFB) table of results

Plant structure	ω (rad/s)	τ (s)	Max actuation	Total actuation	Settle time (s)	Overshoot (%)
1	10	0	1.7212	0.22457	0.1784	0.61067
1	10	0.1	1.4257	0.58452	0.56067	8.25
1	10	0.05	1.8365	0.38207	0.32206	8.7177
1	50	0	1.7212	0.044918	0.035685	0.61067
1	50	0.1	1.1479	0.38212	0.39842	13.2661
1	50	0.05	1.1483	0.22819	0.23443	10.716
2	10	0	199.2938	2.529	0.3046	2.3031
2	10	0.1	50.9481	1.3599	0.63634	8.8128
2	10	0.05	114.9587	2.242	0.25202	8.3084
2	50	0	333.6711	3.1107	0.065618	15.9754
2	50	0.1	40.5438	1.5825	0.69682	21.328
2	50	0.05	59.7381	1.8048	0.38318	21.039
3	10	0	3.4534	0.65981	0.55488	4.902
3	10	0.1	9.9907	0.8442	0.70429	18.0198
3	10	0.05	12.9328	0.53905	0.39502	9.2305
3	50	0	2.2945	0.12968	0.10865	5.2519
3	50	0.1	1.1253	0.38351	0.39139	9.4171
3	50	0.05	1.1381	0.1905	0.18627	7.6357
4	10	0	1.6443	0.27218	0.21915	12.8313
4	10	0.1	1.7413	0.41552	0.36451	16.7991
4	10	0.05	2.3466	0.32315	0.25	26.516
4	50	0	0.99995	2.9595	3.9527	-0.0051387
4	50	0.1	0.99998	2.6544	3.5496	-0.0016679
4	50	0.05	0.99997	2.8112	3.7553	-0.0030613
5	10	0	8.4098	0.47791	0.39104	19.1577
5	10	0.05	13.2699	2.7653	2.0451	157.6398
5	50	0	8.4098	0.095586	0.078212	19.1577
6	10	0	1	1.386	1.8682	0.0014718
6	10	0.1	1.0269	1.9828	2.4505	3.0725
6	10	0.05	1.0018	1.3164	1.8371	0.20327
6	50	0	1	1.2987	1.6725	-4.0536e-10
6	50	0.1	1	1.4947	2.028	-4.3091e-09
6	50	0.05	1	0.9747	1.3301	-3.6593e-10

Table 3: Smith predictor (SP) table of results

Plant structure	ω (rad/s)	τ (s)	Max actuation	Total actuation	Settle time (s)	Overshoot (%)
1	10	0	1.7212	0.22457	0.1784	0.61067
1	10	0.1	1.7212	0.22457	0.1784	0.61067
1	10	0.05	1.7212	0.22457	0.1784	0.61067
1	50	0	1.7212	0.044918	0.035685	0.61067
1	50	0.1	1.7212	0.044918	0.035685	0.61067
1	50	0.05	1.7212	0.044918	0.035685	0.61067
2	10	0	199.2938	2.529	0.3046	2.3031
2	10	0.1	199.2938	2.529	0.3046	2.3031
2	10	0.05	199.2938	2.529	0.3046	2.3031
2	50	0	333.6711	3.1107	0.065619	15.9754
2	50	0.1	333.6711	3.1107	0.065619	15.9754
2	50	0.05	333.6711	3.1107	0.065619	15.9754
3	10	0	3.4534	0.65981	0.55488	4.902
3	10	0.1	3.4534	0.65981	0.55488	4.902
3	10	0.05	3.4534	0.65981	0.55488	4.902
3	50	0	2.2945	0.12968	0.10865	5.2519
3	50	0.1	2.2945	0.12968	0.10865	5.2519
3	50	0.05	2.2945	0.12968	0.10865	5.2519
4	10	0	1.6443	0.27218	0.21915	12.8313
4	10	0.1	1.6443	0.27218	0.21915	12.8313
4	10	0.05	1.6443	0.27218	0.21915	12.8313
4	50	0	0.99995	2.9595	3.9527	-0.0051387
4	50	0.1	0.99995	2.9595	3.9527	-0.0051387
4	50	0.05	0.99995	2.9595	3.9527	-0.0051387
5	10	0	8.4098	0.47791	0.39104	19.1577
5	10	0.05	8.4098	0.47791	0.39104	19.1577
5	50	0	8.4098	0.095586	0.078212	19.1577
6	10	0	1	1.386	1.8682	0.0014718
6	10	0.1	1	1.386	1.8682	0.0014718
6	10	0.05	1	1.386	1.8682	0.0014718
6	50	0	1	1.2987	1.6725	-4.0536e-10
6	50	0.1	1	1.2987	1.6725	-4.0536e-10
6	50	0.05	1	1.2987	1.6725	-4.0536e-10

Table 4: Fully-seperable-degrees-of-freedom (FSDoF) controller table of results

Plant structure	ω (rad/s)	τ (s)	Max actuation	Total actuation	Settle time (s)	Overshoot (%)
1	10	0	1.708	0.22461	0.15501	-1.5787e-11
1	10	0.1	1.4121	0.59165	0.20134	-2.2826e-11
1	10	0.05	1.8227	0.37316	0.14262	-1.5743e-11
1	50	0	1.7055	0.044904	0.031065	-3.9191e-12
1	50	0.1	1.1379	0.39851	0.05807	-7.816e-12
1	50	0.05	1.1384	0.23454	0.058016	-7.7827e-12
2	10	0	199.3839	2.7954	0.13558	-1.6875e-11
2	10	0.1	50.6912	1.3929	0.26331	-2.7456e-11
2	10	0.05	114.3585	2.0797	0.17768	-1.3689e-11
2	50	0	335.1802	1.5862	0.046018	-5.9841e-12
2	50	0.1	40.3913	1	0.12982	-1.7808e-11
2	50	0.05	60.0318	1	0.10649	-1.2257e-11
3	10	0	3.4643	0.64743	0.31345	-3.4639e-11
3	10	0.1	9.9555	0.8409	0.1849	-2.2804e-11
3	10	0.05	12.8506	0.53923	0.16275	-1.7275e-11
3	50	0	2.2862	0.1222	0.077169	-7.1942e-12
3	50	0.1	1.1174	0.39355	0.11039	-1.0014e-11
3	50	0.05	1.1296	0.18863	0.10979	-9.9698e-12
4	10	0	1.6288	0.25412	0.091489	2.2427e-12
4	10	0.1	1.7247	0.40496	0.076459	5.5067e-12
4	10	0.05	2.3247	0.30905	0.022104	2.065e-12
4	50	0	1	3.8393	0.2917	-1.3323e-12
4	50	0.1	1	3.4363	0.2917	-1.3323e-12
4	50	0.05	1	3.642	0.2917	-1.3323e-12
5	10	0	8.3439	0.44429	0.028242	-2.8422e-12
5	10	0.05	13.1447	2.1131	0.017309	-2.3537e-12
5	50	0	8.3347	0.088853	0.0056623	-1.2212e-13
6	10	0	1	1.746	0.81366	-1.5312e-10
6	10	0.1	1	2.3283	0.81366	-1.5312e-10
6	10	0.05	1	1.7149	0.81366	-1.5312e-10
6	50	0	1	1.6516	0.18295	-1.7124e-10
6	50	0.1	1	2.0072	0.18295	-1.7124e-10
6	50	0.05	1	1.3093	0.18295	-1.7124e-10
